# Rasa3 deficiency minimally affects thrombopoiesis but promotes severe thrombocytopenia due to integrin-dependent platelet clearance

**DOI:** 10.1172/jci.insight.155676

**Published:** 2022-04-22

**Authors:** Robert H. Lee, Dorsaf Ghalloussi, Gabriel L. Harousseau, Joseph P. Kenny, Patrick A. Kramer, Fabienne Proamer, Bernhard Nieswandt, Matthew J. Flick, Christian Gachet, Caterina Casari, Anita Eckly, Wolfgang Bergmeier

**Affiliations:** 1 Department of Biochemistry and Biophysics and; 2 Blood Research Center, School of Medicine, University of North Carolina at Chapel Hill, North Carolina, USA.; 3 Université de Strasbourg, INSERM, EFS Grand Est, BPPS UMR-S 1255, FMTS, F-67065 Strasbourg, France.; 4 Institute of Experimental Biomedicine I, University Hospital Würzburg, Würzburg, Germany.; 5 Rudolf Virchow Center, University of Würzburg, Würzburg, Germany.; 6 Department of Pathology and Laboratory Medicine, School of Medicine, University of North Carolina at Chapel Hill, North Carolina, USA.; 7 HITh, UMR_S1176, INSERM, Université Paris-Saclay, Le Kremlin-Bicêtre, France.

**Keywords:** Cell Biology, Hematology, G proteins, Integrins, Platelets

## Abstract

Platelet homeostasis is dependent on a tight regulation of both platelet production and clearance. The small GTPase Rap1 mediates platelet adhesion and hemostatic plug formation. However, Rap1 signaling is also critical for platelet homeostasis as both Rap1 deficiency and uninhibited Rap1 signaling lead to marked thrombocytopenia in mice. Here, we investigated the mechanism by which deficiency in Rasa3, a critical negative regulator of Rap1, causes macrothrombocytopenia in mice. Despite marked morphological and ultrastructural abnormalities, megakaryocytes in hypomorphic *Rasa3^hlb/hlb^* (*R3^hlb/hlb^*) or *Rasa3^–/–^* mice demonstrated robust proplatelet formation in vivo, suggesting that defective thrombopoiesis is not the main cause of thrombocytopenia. Rather, we observed that *R3^hlb/hlb^* platelets became trapped in the spleen marginal zone/red pulp interface, with evidence of platelet phagocytosis by macrophages. Clearance of mutant platelets was also observed in the liver, especially in splenectomized mice. Platelet count and platelet life span in Rasa3-mutant mice were restored by genetic or pharmacological approaches to inhibit the Rap1/talin1/α_IIb_β_3_ integrin axis. A similar pattern of splenic clearance was observed in mice injected with anti-α_IIb_β_3_ but not anti–glycoprotein Ibα platelet-depleting antibodies. In summary, we describe a potentially novel, integrin-based mechanism of platelet clearance that could be critical for our understanding of select inherited and acquired thrombocytopenias.

## Graphical abstract

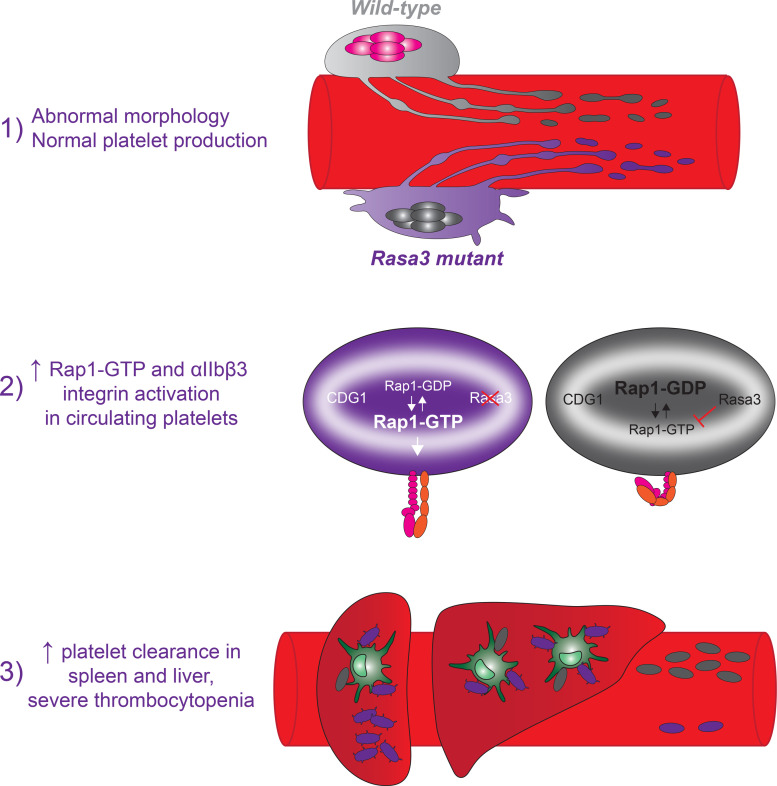

## Introduction

Hemostasis requires tight regulation of both platelet production and function. Billions of platelets are produced each day; they are released into the bloodstream by megakaryocytes (MKs) in bone marrow (BM) (1) and lung (2) and circulate quiescently to monitor the vasculature until they are either consumed during hemostasis or cleared at the end of a well-defined life span. Normally, platelet life span (approximately 5 days in mice and approximately 10 days in humans) is regulated by an “internal clock” involving a balancing act between prosurvival and proapoptotic Bcl family proteins (3). Physiological platelet clearance is thought to be regulated mainly by desialylation of major platelet surface glycoproteins (GPs), primarily GPIbα, leading to clearance in the liver by resident intravascular macrophages, Kupffer cells (KCs), and hepatocytes (4, 5). Other mechanisms such as phosphatidylserine (PS) exposure are thought to contribute to the clearance of old platelets, but this has yet to be demonstrated in vivo (6). Conversely, pathological platelet clearance and thrombocytopenia are observed in numerous disease states, including immune thrombocytopenia (ITP) (7), type 2B and platelet type von Willebrand’s disease (8, 9), viral infections such as Dengue fever (10), and sepsis (11), among others.

Platelets are able to adhere and activate in the shear environment of the vasculature via a series of ligand-receptor interactions and intracellular signaling events. Platelets bind to von Willebrand factor and collagen via the GPIb-IX-V complex and GPVI, respectively, both of which induce intracellular signaling (12). Thrombin, generated at the site of injury, potently activates platelets through protease-activated receptors 1 and 4. ADP and thromboxane A_2_ released from activated platelets then act to reinforce the hemostatic plug by providing secondary stimulation. While GPIbα mediates platelet tethering to the site of vascular damage, α_IIb_β_3_ integrin (GPIIbIIIa) is required for platelet-platelet cohesion and aggregation (13). The small GTPase Rap1 is a critical regulator of α_IIb_β_3_ activation (14, 15) and undergoes biphasic activation (16). Following platelet stimulation, increased cytosolic Ca^2+^ causes activation of the guanine nucleotide exchange factor (GEF), CalDAG-GEFI, which mediates rapid nucleotide exchange on Rap1 (17). Sustained Rap1 activation requires inhibition of the GAP Rasa3, which normally enhances GTP hydrolysis on Rap1 to prevent activation of circulating platelets (18). The signal for Rasa3 inhibition is provided by ADP binding to the G_i_-coupled receptor, P2Y12.

Loss of CalDAG-GEFI causes platelet dysfunction and moderate to severe bleeding in mice (19) and humans (20). Deletion of the major platelet Rap1 isoform, Rap1b, similarly impairs platelet function and increases bleeding in mice (14). Simultaneous loss of Rap1a and Rap1b further prolongs bleeding times; it also causes macrothrombocytopenia due to impaired platelet production (15). In comparison, mutant mice with impaired Rasa3 expression or function are severely thrombocytopenic (18, 21, 22). Ex vivo studies in mice expressing a catalytically inactive Rasa3 variant (22) (Rasa3^ΔGAP^) or a point mutant (21) (G125V) focused on defective MK function as the cause of the observed thrombocytopenia. Both alterations in MK ultrastructure and a defect in proplatelet formation (PPF) were reported. Utilizing mice with another missense mutation in Rasa3 (H794L; *Rasa3^hlb/hlb^* [*R3^hlb/hlb^*]), we reported higher baseline levels of Rap1-GTP and premature clearance of circulating mutant platelets (18). Concomitant loss of CalDAG-GEFI (*Cdg1^–/–^*) normalized both platelet count and platelet life span in *R3^hlb/hlb^* mice, suggesting that unrestrained Rap1 activation leading to enhanced platelet clearance is the main reason for thrombocytopenia in Rasa3-mutant mice.

In this study, we aimed to definitively determine whether the severe macrothrombocytopenia in Rasa3-mutant mice is predominantly the result of defective platelet production or impaired platelet survival and how Rasa3-mutant platelets are prematurely cleared from circulation. Our in-depth analysis of MK function demonstrates that, despite evidence of abnormal MK morphology, in vivo platelet production is largely normal in *R3^hlb/hlb^* and *Rasa3^–/–^* (*R3^–/–^*) mice. Once released into circulation, Rasa3-mutant platelets became rapidly trapped in the peri–marginal zone (peri-MZ) of the spleen, in both a macrophage-independent and -dependent manner. Enhanced macrophage-dependent clearance of mutant platelets was also observed in the liver, especially in splenectomized mice. Platelet count was partially restored in *R3^hlb/hlb^* mice by targeting talin1 or α_IIb_β_3_ integrin. Finally, we demonstrated that anti-α_IIb_β_3_ antibodies induced platelet clearance in the spleen in a remarkably similar pattern to Rasa3-mutant mice. Our results suggest what we believe to be a novel, integrin-dependent clearance mechanism for hyperreactive platelets, which may contribute to low platelet counts observed in some inherited and acquired thrombocytopenias.

## Results

### R3^hlb/hlb^ MKs show abnormal morphology and ultrastructure in situ.

**Figure 1:**
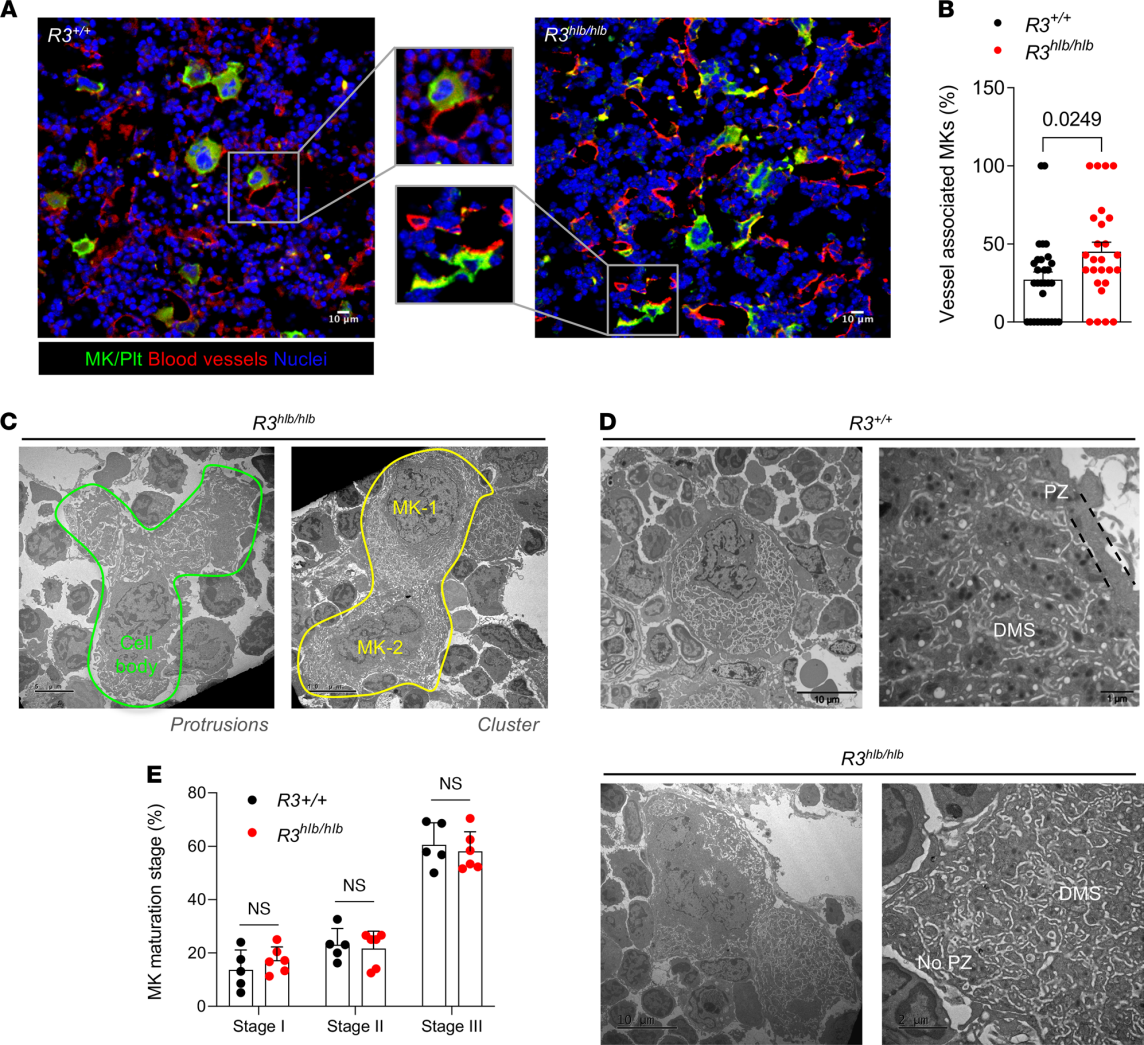
*R3^hlb/hlb^* MKs show abnormal morphology and ultrastructure in situ. (**A**) BM cryosections from control (*R3^+/+^*) and *R3^hlb/hlb^* mice were immunostained for MKs/platelets with anti–GPIX-AF488 (green), sinusoids with anti–CD105-AF647 (red), and nuclei with Hoechst (blue), and imaged by confocal microscopy. Boxes indicate localization of enlarged images (original magnification, ×200) shown in the middle panel. Scale bars: 10 μm. (**B**) Quantification of MKs in direct contact with sinusoids in the BM of *R3^+/+^* and *R3^hlb/hlb^* mice (*n* = 26–30 fields from a total of 5 mice per group). (**C**) Representative transmission electron microscopy (TEM) images of *R3^hlb/hlb^* MKs forming protrusions (green outline) and forming clusters of multiple MKs (yellow outline). Scale bars: 5 μm (left), 10 μm (right). (**D**) Representative TEM images of MKs from *R3^+/+^* (top panels) and *R3^hlb/hlb^* mice (bottom panels) showing structure of DMS and PZ; overview (left panels; scale bar: 10 μm) and detail (right panels; scale bar: 1–2 μm). (**E**) Maturation stage distribution (I–III; see Methods) of differentiated BM MKs (*n* = 5–6). Data shown as mean ± SEM. Statistical significance was determined using unpaired 2-tailed Student’s *t* test.

We first investigated whether the severe macrothrombocytopenia in *R3^hlb/hlb^* mice (29 × 10^6^/mL vs. 1039 × 10^6^/mL in controls; ref. 18) is caused by a defect in platelet production. We previously reported increased numbers of MKs in H&E-stained BM and spleen sections in *R3^hlb/hlb^* mice (18). BM cryosections immunostained for MKs (GPIX/CD42a) and blood vessels (endoglin/CD105) revealed marked morphological alterations in the mutant MKs. Compared to *Rasa3^+/+^* (*R3^+/+^*) MKs, which mostly exhibited a rounded morphology, *R3^hlb/hlb^* BM MKs showed irregular cytoplasmic contours and an abnormal shape (Figure 1A). Some *R3^hlb/hlb^* MKs appeared very large, although the marked morphological abnormalities precluded us from accurately measuring the size of individual MKs. Moreover, significantly more *R3^hlb/hlb^* MKs were in direct contact with sinusoidal blood vessels (Figure 1B). 3D renderings generated from confocal *Z*-stacks of immunostained BM whole mounts illustrate the full extent of the morphological abnormalities in mutant MKs (Supplemental Videos 1 and 2; supplemental material available online with this article; https://doi.org/10.1172/jci.insight.155676DS1). Electron microscopy (EM) studies identified long MK protrusions within the BM compartment in *R3^hlb/hlb^* samples and *R3^hlb/hlb^* MKs frequently appeared in clusters (Figure 1C). EM images also provided critical information on the ultrastructure of the mutant MKs, including the demarcation membrane system (DMS) and the peripheral zone (PZ) that delimits the DMS and prevents premature PPF (23, 24). While the DMS network in both *R3^+/+^* and *R3^hlb/hlb^* MKs was normally distributed, the PZ in *R3^hlb/hlb^* MKs appeared thin or lacking (Figure 1D). However, no difference in the maturation stage distribution (I–III; see Methods) of differentiated BM MKs was observed between control and *R3^hlb/hlb^* mice (Figure 1E).

### PPF by R3^hlb/hlb^ MKs is impaired ex vivo but normal in vivo.

**Figure 2:**
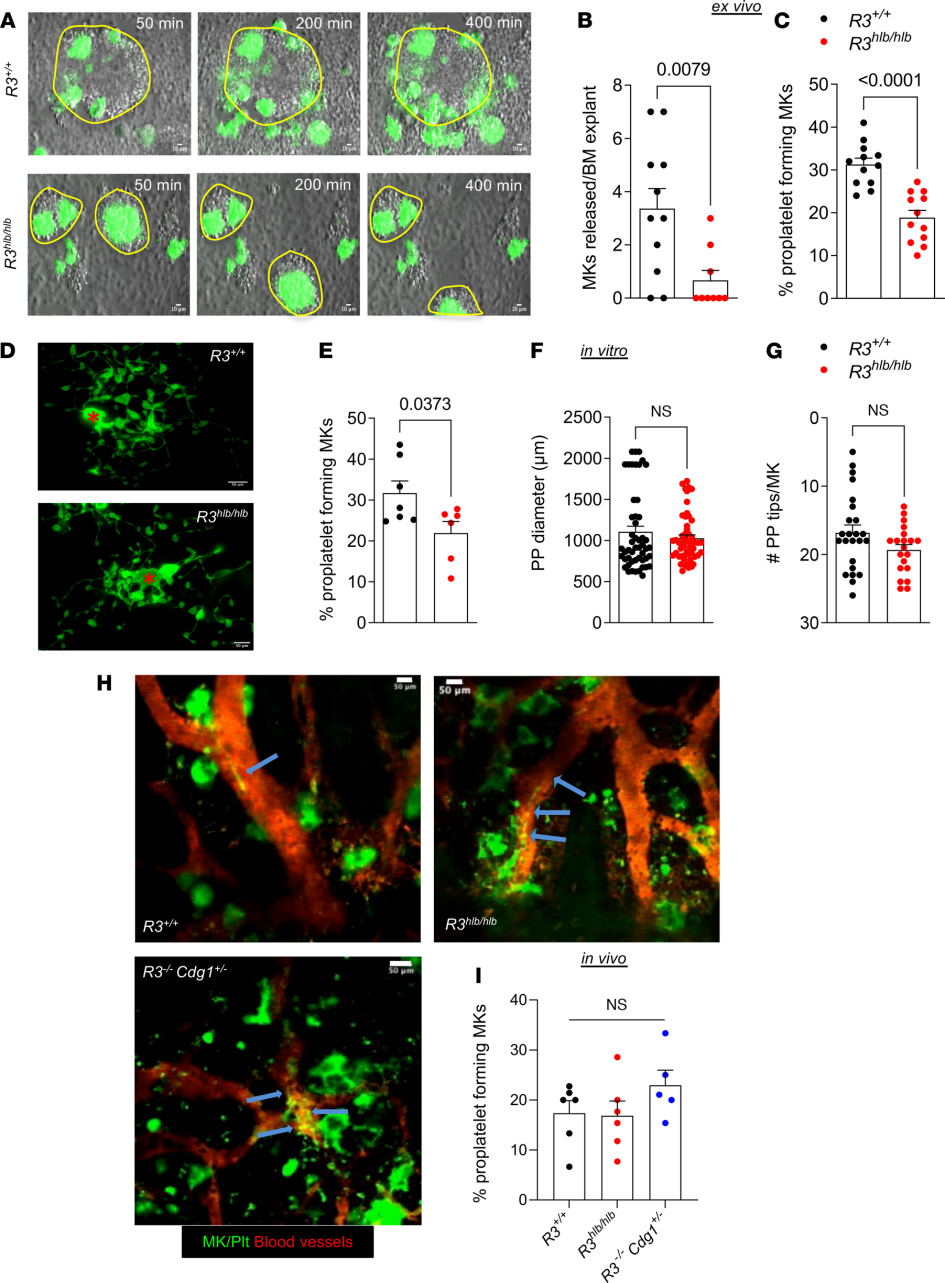
PPF by *R3^hlb/hlb^* MKs is impaired ex vivo but normal in vivo. (**A**) Representative images of ex vivo proplatelet-forming MKs from *R3^+/+^* (upper panels) and *R3^hlb/hlb^* mice (lower panels) taken at the indicated time points after start of imaging. Samples were stained with anti–GPIX-AF488 to label MKs/platelets (green). Scale bars: 10 μm. Yellow lines delimit BM pieces. (**B**) MKs released from BM explants, expressed as number of MKs released per explant in each field of view (*n* = 9–11). (**C**) Proplatelet-forming MKs in BM explants from *R3^+/+^* and *R3^hlb/hlb^* mice, expressed as percentage of total MKs per field of view (*n* = 12). (**D**) Representative images of in vitro proplatelet-forming MKs from *R3^+/+^* (top) and *R3^hlb/hlb^* (bottom) mice. Scale bars: 50 μm. Red asterisk marks the MK cell body. (**E**) Percentage of proplatelet-forming MKs from *R3^+/+^* and *R3^hlb/hlb^* mice in vitro (*n* = 6–7). (**F** and **G**) Quantification of the proplatelet (PP) diameter (μm) (*n* = 50 MKs) and number of PP tips per MK (*n* = 20–24). (**H**) Representative still frames from calvarial BM 2P-IVM videos showing PPF in *R3^+/+^*, *R3^hlb/hlb^*, and *R3^–/–^* *Cdg1^+/–^* mice. Vessels were visualized by i.v. administration of tetramethylrhodamine-dextran (red), and MKs/platelets were labeled with anti–GPIX-AF488 antibodies (green). Scale bars: 50 μm. Blue arrows point to PP extensions within blood vessels. (**I**) Quantification of in vivo PPF by MKs in BM of *R3^+/+^*, *R3^hlb/hlb^*, and *R3^–/–^ Cdg1^+/–^* mice expressed as percentage of total MKs per field of view (*n* = 5–6 mice). Data shown as mean ± SEM. Statistical significance was determined using unpaired 2-tailed Student’s *t* test in **B**, **C**, and **E**–**G** or 1-way ANOVA in **I**.

We next investigated whether the morphological and structural abnormalities in *R3^hlb/hlb^* MKs affect PPF. To assess PPF within the BM environment, we performed an ex vivo PPF assay using intact BM explants and quantified both the number of MKs released from the BM explant and the percentage of MKs undergoing PPF. In this assay, MKs first move to the periphery of the BM pieces before they are able to be visualized undergoing PPF (25). While control MKs effectively escaped the BM and underwent PPF, *R3^hlb/hlb^* MKs were trapped within the BM pieces and fewer MKs underwent PPF (Figure 2, A–C, and Supplemental Videos 3 and 4). These results are consistent with previous findings in MKs from mice expressing a catalytically dead variant of Rasa3, which exhibited increased adhesion to matrix components and markedly impaired PPF in a similar ex vivo assay (22). When analyzed in vitro, i.e., when MKs were resuspended from BM as single cells prior to the PPF assay (Figure 2D), *R3^hlb/hlb^* MKs demonstrated a reduction in PPF (Figure 2E), but the diameter of proplatelets (Figure 2F) and the number of proplatelet tips per MK (Figure 2G) were normal compared with control MKs. To determine whether *R3^hlb/hlb^* MKs are impaired in their ability to undergo PPF in the mouse, we quantified PPF in live mice using 2-photon intravital microscopy (2P-IVM) in the calvarial BM, as previously described (26). These studies confirmed our ex vivo observations of abnormal morphology and clustering of MKs in *R3^hlb/hlb^* mice (Figure 2H). However, the percentage of MKs undergoing PPF was normal when compared with control mice (Figure 2I). Similar observations were made in *R3^–/–^ Cdg1^+/–^* mice completely lacking Rasa3 expression (Figure 2, H and I), which also exhibit severe thrombocytopenia and a marked reduction in platelet life span (Supplemental Figure 1). *R3^–/–^* mice that express both alleles of Cdg1 are not viable (18) and, thus, could not be analyzed.

### R3^hlb/hlb^ platelets become trapped at the MZ/red pulp interface in the spleen and on KCs in the liver.

**Figure 3:**
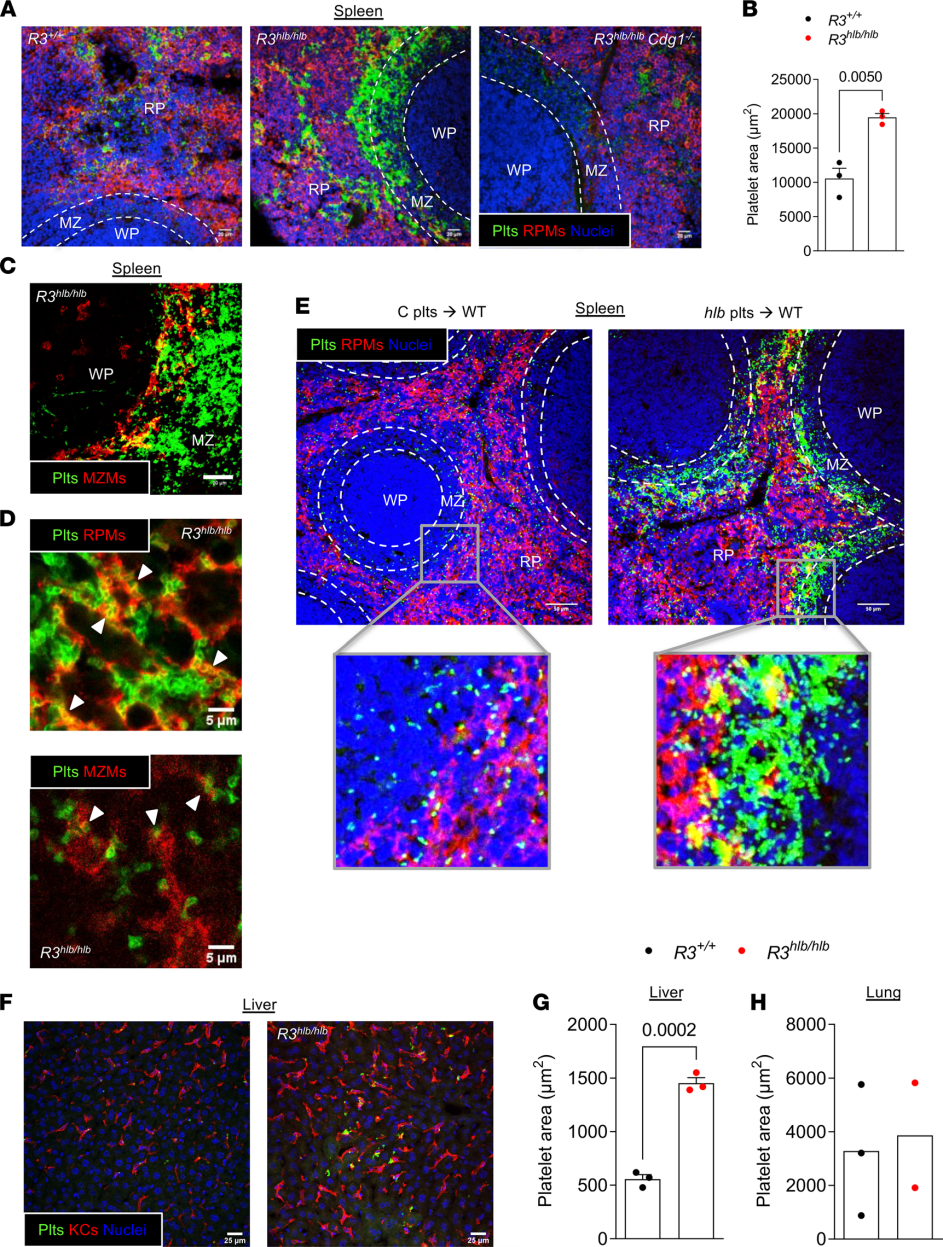
*R3^hlb/hlb^* platelets become trapped at the MZ/red pulp interface in the spleen and on KCs in the liver. (**A**) Confocal immunofluorescence (IF) images of spleen cryosections from the indicated mice, stained for platelets with anti–GPIX-AF488 (green), RPMs with anti–F4/80-AF647 (red), and nuclei with Hoechst (blue). Morphologically distinct areas are marked as white pulp (WP), MZ, and RP and defined by dashed lines. Scale bars: 20 μm. (**B**) Quantification of platelet area in spleen cryosections (*n* = 3). (**C**) IF image of *R3^hlb/hlb^* spleen cryosection stained for platelets (green) and MZMs with anti–CD209b + anti–hamster-AF647 (red). Scale bar: 20 μm. (**D**) IF images of *R3^hlb/hlb^* spleen cryosections stained for platelets (green) and macrophages (RPMs on top, MZMs on bottom; red). White arrowheads denote probable phagocytosis of platelets by macrophages. Scale bars: 5 μm. (**E**) IF images of cryosections from spleens obtained from WT recipient mice, 24 hours after transfusion with EGFP-expressing *R3^+/+^ Cdg1^+/–^* (C) or *R3^hlb/hlb^ Cdg1^+/–^* (*hlb*) platelets, stained for RPMs (red) and nuclei (Hoechst, blue). Transfused platelets are visualized by EGFP fluorescence. Boxes indicate localization of enlarged images (original magnification, ×100) (bottom panels). Scale bars: 50 μm. (**F**) IF images of liver cryosections stained for platelets (green), KCs, anti–F4/80-AF647 (red), and nuclei (blue). Scale bars: 25 μm. (**G** and **H**) Quantification of platelet area in liver and lung cryosections (*n* = 2–3). Data shown as mean ± SEM. Statistical significance was determined using unpaired 2-tailed Student’s *t* test.

Our in vivo studies provide evidence that platelet production is largely intact in Rasa3-mutant mice despite marked MK abnormalities. Consequently, increased platelet clearance is likely the main cause of the severe thrombocytopenia observed in these mice. We previously found that radiolabeled *R3^hlb/hlb^* platelets predominantly accumulate in the spleen and to a lesser degree in the liver (18), so we first sought to determine the mechanism of splenic clearance of *R3^hlb/hlb^* platelets. Spleen cryosections from *R3^hlb/hlb^* and control mice were immunostained for platelets (GPIX) and red pulp macrophages (RPMs; F4/80). In control mice, single or small groups of platelets were mostly found throughout the red pulp (RP), the F4/80-positive areas of the spleen (Figure 3A). In stark contrast, large accumulations of platelets were found at the interface of the MZ and RP in spleens from *R3^hlb/hlb^* mice, with platelets also observed within the RP (Figure 3A). Quantification of platelet area in spleen sections demonstrated significantly greater platelet accumulation in *R3^hlb/hlb^* spleens compared with *R3^+/+^* mice (Figure 3B). Platelets could be seen in contact with RPMs but also in RPM-free areas. Platelet accumulation was not observed in the lymphocyte-rich white pulp (F4/80-negative areas with closely packed nuclei) in any genotype. Concomitant deletion of CalDAG-GEFI in *R3^hlb/hlb^* mice prevented the accumulation of mutant platelets within the splenic peri-MZ (Figure 3A), while *R3^hlb/hlb^ Cdg1^+/–^* mice had a phenotype more similar to *R3^hlb/hlb^* mice (not shown). Marginal zone macrophages (MZMs) are a subset of macrophages that delimit the splenic MZ and are some of the first macrophages to encounter foreign organisms entering the spleen (27). When we coimmunostained *R3^hlb/hlb^* spleen cryosections for platelets and MZMs (CD209b), we observed partial colocalization of platelets with MZMs (Figure 3C). In fact, we observed many *R3^hlb/hlb^* platelets in contact with or surrounded by macrophage membrane, both RPM and MZM (Figure 3D), suggesting ongoing phagocytosis of trapped *R3^hlb/hlb^* platelets by spleen macrophages.

To track the clearance of mutant platelets more effectively, we performed adoptive transfer studies with EGFP-expressing *R3^hlb/hlb^ Cdg1^+/–^* (*hlb*) and *R3^+/+^ Cdg1^+/–^* control (C) platelets into WT mice. Compared with C platelets, significantly fewer *hlb* platelets remained in circulation 24 hours after transfusion into recipient mice (Supplemental Figure 2), and similar to our findings with endogenous platelets, large numbers of transfused *hlb* platelets accumulated primarily at the splenic MZ/RP interface (Figure 3E). 2P-IVM studies showed that transfused *hlb* platelets were firmly adhered in the RP of recipient mice, while most C platelets only made transient contacts (Supplemental Videos 5 and 6). However, only a small fraction of *hlb* platelets appeared to be in direct contact with F4/80-positive macrophages (Supplemental Video 6), suggesting that platelets are initially trapped in the spleen in a macrophage-independent manner. This is further supported by the observation that *hlb* platelets still accumulate at the MZ/RP interface in mice treated with clodronate liposomes (28) to deplete spleen macrophages (Supplemental Figure 3).

Despite the strong evidence that *R3^hlb/hlb^* platelets are primarily cleared in the spleen, splenectomy did not improve platelet count in these mice (18). When we immunostained liver sections, we found almost 3 times the number of *R3^hlb/hlb^* platelets cleared in the liver (Figure 3, F and G), though the absolute platelet area was far less than that observed in spleen sections. Almost all platelets in both genotypes were found to be in contact with KCs, and clusters of *R3^hlb/hlb^* platelets could be seen (Figure 3F). No difference between genotypes was observed in lung sections (Figure 3H). To investigate platelet clearance in the liver of splenectomized mice, we performed platelet-tracking experiments with transfused EGFP^+^ C and *hlb* platelets. In splenectomized recipient mice, we observed a delay in very early clearance (<30 minutes) of transfused *hlb* platelets (Supplemental Figure 4A). However, the rate of transfused platelet clearance in splenectomized mice was similar to sham-operated recipients at 24 and 48 hours after transfusion (Supplemental Figure 4B). When we investigated liver clearance of transfused *hlb* platelets using tissue staining and liver confocal intravital microscopy, we found more *hlb* platelets being cleared in the liver in the absence of the spleen (Supplemental Figure 4, C–E, and Supplemental Videos 7 and 8). Transfused *hlb* platelets were also found trapped in the lungs of splenectomized recipient mice (Supplemental Figure 4F). These results suggest that multiple mechanisms and tissues ensure the clearance of hyperreactive platelets from circulation.

### Increased α_IIb_β_3_ integrin activation is the only abnormal clearance marker on R3^hlb/hlb^ platelets.

**Figure 4:**
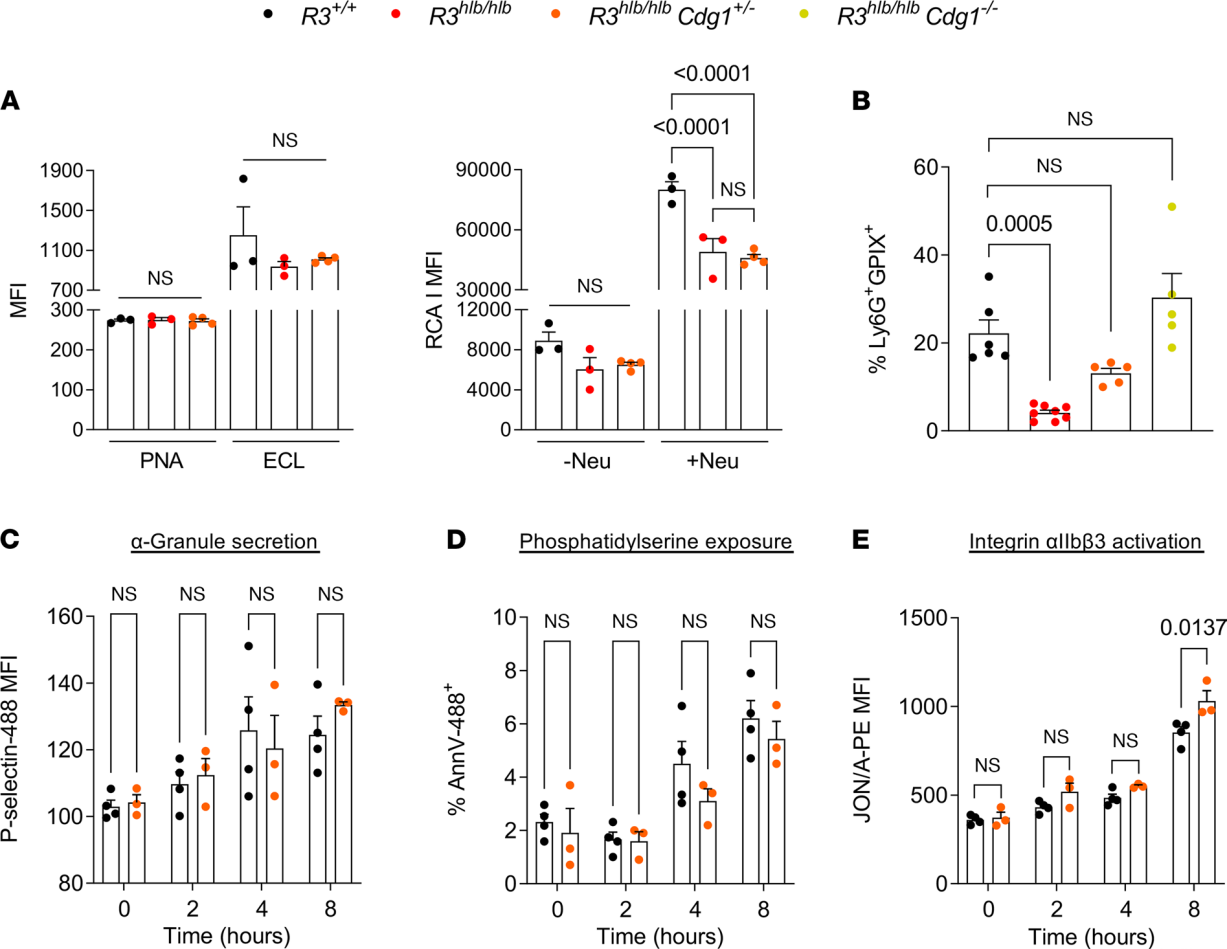
Increased α_IIb_β_3_ integrin activation is the only abnormal clearance marker on *R3^hlb/hlb^* platelets. (**A**) Platelet receptor glycosylation was determined in vitro by binding of FITC-conjugated lectins (PNA, ECL, RCA1) and analyzed by flow cytometry. Pretreatment of platelets with neuraminidase (Neu; 100 U/mL) was used as a positive control for desialylation (RCA1) (*n* = 3–4). (**B**) PNAs were determined in whole blood. Diluted blood samples were incubated with anti-Ly6G (neutrophils) and anti-GPIX (platelets) antibodies and analyzed by flow cytometry following RBC lysis and fixation. PNAs were identified as GPIX^+^ cells after gating on Ly6G expression (*n* = 5–8). (**C**–**E**) Heparinized whole blood from mice was left on the bench for 8 hours at room temperature (RT), and markers of platelet activation and apoptosis (P selectin, PS exposure, and activated α_IIb_β_3_ integrin) were determined every 2 hours by flow cytometry (*n* = 3–4). Data shown as mean ± SEM. Statistical significance was determined using 1-way ANOVA in **A** and **B** or 2-way ANOVA in **C**–**E** with Bonferroni’s correction for multiple comparisons.

The formation of autoantibodies and changes in the glycosylation of platelet surface receptors are well established triggers for platelet clearance. Our adoptive transfer studies effectively ruled out autoimmunity as the trigger for increased platelet turnover in Rasa3-mutant mice, and *R3^hlb/hlb^* platelets did not exhibit increased exposure of galactosyl (β-1,4) N-acetylglucosamine (Galβ4GlcNAc) (Erythrina cristagalli lectin, ECL) or galactosyl (β-1,3) N-acetylgalactosamine (Galβ3GalNAc) (peanut agglutinin, PNA) residues (Figure 4A; ref. 29). In fact, *R3^hlb/hlb^* platelets exhibited reduced Ricinus communis agglutinin I (RCA1) lectin binding (exposed galactose residues, Galβ1-4GlcNAc), even after treatment with neuraminidase (Figure 4A), suggesting greater sialic acid content. Rap1 signaling controls various platelet responses, including integrin activation, secretion, and PS exposure (30). Increased secretion may lead to surface expression of P selectin and the formation of PNAs (31). However, the number of PNAs was lower in blood of Rasa3-mutant mice, consistent with their lower peripheral platelet count, and circulating PNAs increased back to control levels in *R3^hlb/hlb^ Cdg1^–/–^* mice with normal platelet counts (Figure 4B). Additionally, we did not observe increased surface exposure of P selectin or PS on freshly isolated or in vitro aged *R3^hlb/hlb^* platelets (Figure 4, C and D). Increased α_IIb_β_3_ integrin activation was the only significant difference observed between in vitro aged *R3^hlb/hlb^ Cdg1^+/–^* and *R3^+/+^* platelets (Figure 4E).

### Clearance of Rasa3-mutant platelets is mediated in part by talin1 and α_IIb_β_3_ integrin.

**Figure 5:**
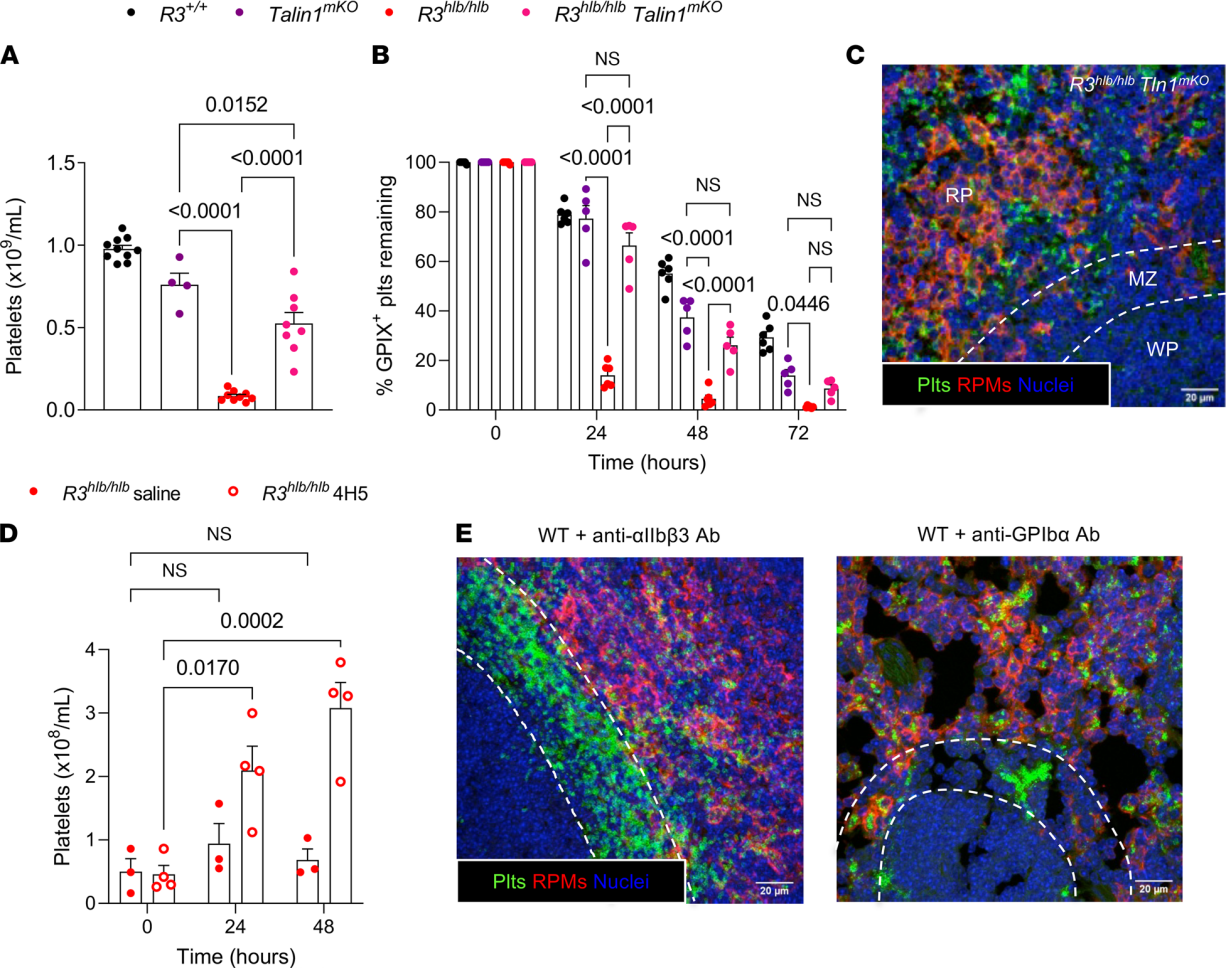
Clearance of Rasa3-mutant platelets in the spleen is mediated in part by talin1 and α_IIb_β_3_ integrin. (**A**) Circulating platelet counts were determined by flow cytometry using whole blood from *R3^hlb/hlb^*, *Talin1^fl/fl^ Pf4-Cre^+^* (*Tln1^mKO^*), or *R3^hlb/hlb^* *Tln1^mKO^* double-mutant mice (*n* = 4–10). (**B**) Mice were injected i.v. with anti–GPIX-AF488 antibody (2.5 μg/mouse) on day 0 to label all circulating platelets, and then the percentage of GPIX-AF488^+^ platelets remaining was determined every 24 hours by flow cytometry (*n* = 5–6). (**C**) IF image of a *R3^hlb/hlb^* *Tln1^mKO^* spleen cryosection stained for platelets with anti–GPIX-AF488 (green), RPMs with anti–F4/80-AF647 (red), and nuclei with Hoechst (blue). Scale bar: 20 μm. (**D**) *R3^hlb/hlb^* mice were injected once per day with a blocking anti–α_IIb_β_3_ F(ab′)_2_ antibody (clone 4H5, 75 μg) or saline, and platelet counts were determined before (*t* = 0) and after (*t* = 24, 48 hours) injection (*n* = 3–4). (**E**) IF images of spleen cryosections collected from WT mice 30 minutes after bolus injection with anti-α_IIb_β_3_ (clone MWReg30) or anti-GPIbα (clone R300) platelet-depleting antibodies (2 μg/g BW), demonstrating peri-MZ accumulation of platelets with anti-α_IIb_β_3_ but not anti-GPIbα antibodies. Cryosections were stained for platelets (green), RPMs (red), and nuclei (blue). Scale bar: 20 μm. Data shown as mean ± SEM. Statistical significance was determined using 1-way ANOVA in **A** or 2-way ANOVA in **B** and **D** with Bonferroni’s correction for multiple comparisons.

To determine whether integrin activation contributes to the clearance of Rasa3-mutant platelets, we crossed *R3^hlb/hlb^* mice with MK/platelet-specific talin1-KO (*Tln^mKO^*) mice (32) and analyzed platelet counts and platelet survival in double-mutant mice. Both platelet count and platelet survival in peripheral blood were significantly improved in the double-mutant mice compared with *R3^hlb/hlb^* mice (Figure 5, A and B). No splenic platelet trapping at the MZ/RP interface was observed in *R3^hlb/hlb^ Tln^mKO^* mice (Figure 5C). Additionally, deletion of talin1 in *R3^hlb/hlb^* mice normalized MK release from BM explants (Supplemental Figure 5) and partially normalized the altered MK morphology (Supplemental Video 9). A significant increase in the peripheral platelet count was also observed in *R3^hlb/hlb^* mice treated with F(ab′)_2_ fragments of a blocking antibody of α_IIb_β_3_ (Figure 5D), suggesting the effect of talin1 deficiency is in part due to impaired α_IIb_β_3_ activation. However, *R3^hlb/hlb^* platelets were cleared as fast as C platelets when injected into fibrinogen γ^Δ*5/*Δ5^ mice lacking a critical binding site for α_IIb_β_3_ (33) (Supplemental Figure 6). Therefore, the α_IIb_β_3_/fibrinogen interaction required for platelet aggregation is not involved in mediating *R3^hlb/hlb^* platelet clearance.

Our mechanistic studies with Rasa3-mutant platelets suggest that premature α_IIb_β_3_ activation in circulation leads to sequestration of platelets at the peri-MZ in the spleen. ITP can be caused by circulating antibodies against α_IIb_β_3_ in humans (34) and mice (35). We therefore asked whether anti-α_IIb_β_3_ antibodies cause platelet clearance by a mechanism similar to that observed in Rasa3-mutant mice. Consistent with our hypothesis, platelets in WT mice injected with a bolus of anti-α_IIb_β_3_ antibody were sequestered at the MZ/RP interface in the spleen (Figure 5E). In contrast, injection of WT mice with antibodies against GPIbα caused platelet clumping and clearance primarily in the liver, though some phagocytosis of platelets was observed in the spleen (Figure 5E). These results suggest that antibodies against α_IIb_β_3_ may cause rapid thrombocytopenia in part due to macrophage-independent platelet sequestration in the spleen and that the potentially novel mechanism of platelet clearance observed in *R3^hlb/hlb^* mice may be relevant to human disease.

## Discussion

Platelet-mediated hemostasis has several absolute requirements to function normally: a) consistent production and release of platelets from MKs into the bloodstream to maintain circulating platelet counts; b) quiescence of circulating platelets to avoid unwanted adhesion or clearance; and c) a rapid, near-immediate platelet response upon vascular injury to form a hemostatic plug. Signaling by the small GTPase Rap1 is critical for platelet production and function (14, 15). In mice lacking the Rap1-GAP Rasa3, which maintains Rap1 in the GDP-bound “off” state, uninhibited Rap1 activity leads to premature platelet activation and a dramatic reduction in the circulating platelet count (18). Here we used various mouse models and approaches to demonstrate that macrothrombocytopenia associated with Rasa3 deficiency is predominantly caused by integrin-mediated platelet clearance. Rasa3-mutant MKs exhibit abnormal morphology and increased adhesiveness to BM sinusoids, but these abnormalities did not significantly reduce the occurrence of PPF in vivo. Once released into circulation, Rasa3-mutant platelets are rapidly sequestered in the spleen at the MZ/RP interface and subsequently phagocytosed by resident macrophages. *R3^hlb/hlb^* platelets are also cleared to a greater extent by the liver, and this clearance mechanism is further enhanced upon splenectomy. Premature clearance of Rasa3-mutant platelets is dependent in part on talin1 and α_IIb_β_3_ integrin, and antibodies against α_IIb_β_3_ cause platelets to be cleared in the spleen in a similar manner to Rasa3-mutant platelets. These findings uncover a potentially novel mechanism of integrin-dependent platelet clearance different from overt thrombosis.

Recent studies identified the Rap1-GAP, Rasa3, as a critical negative regulator of Rap1 signaling in platelets and MKs (18, 21, 22). Thrombocytopenia in Rasa3-mutant mice was assigned to both increased platelet clearance and impaired platelet production. However, no study analyzed both processes in the same mutant strains, and only platelet survival was assessed in vivo. Consistent with findings in Rasa3^ΔGAP^ chimeric mice (22), we observed a) altered MK morphology, b) increased MK adhesiveness to the BM vasculature, and c) impaired PPF ex vivo for *R3^hlb/hlb^* MKs. However, proplatelet characteristics were largely normal in *R3^hlb/hlb^* MKs when cultured as single cells, and, importantly, occurrence of PPF in the BM was comparable between *R3^hlb/hlb^* or *R3^–/–^* *Cdg1^+/–^* and control mice. Currently, we are only equipped to quantify the number of BM MKs undergoing PPF in vivo and cannot rule out differences in the dynamics of release or quality of proplatelets. Neither did we assess PPF in the spleen or lung in this study. Nevertheless, our work provides what we believe is the first in vivo evidence that uninhibited Rap1 signaling does not markedly impair PPF in the BM of mice. We have previously shown that mice deficient in both Rap1a and Rap1b exhibit a marked macrothrombocytopenia due to a defect in PPF (15) and that this activity of Rap1 is not dependent on its ability to bind talin and activate integrins (36). Thus, it may not be surprising that PPF is intact in Rasa3-mutant mice, considering that this GAP primarily controls Rap1 signaling linked to integrin activation (18). Future studies will need to address which Rap-GAP(s) are critical for regulating PPF in MKs.

A key conclusion of this work is that increased platelet clearance is the main reason for thrombocytopenia in Rasa3-mutant mice. Based on the current understanding of platelet clearance mechanisms (37) and the role of Rap1 as a master regulator of platelet function (30), we considered that changes in platelet apoptosis (3), receptor glycosylation (4), or PNA formation (38) could be responsible for the increased turnover of Rasa3-mutant platelets. However, no difference in platelet receptor glycosylation or increased PS exposure was observed in these cells when compared with controls, and mutant mice had reduced PNAs correlating with their low circulating platelet count. One caveat to the analysis of blood platelets is that we are only analyzing those platelets remaining in circulation, and it is possible that expression of clearance markers causes rapid removal of platelets from circulation. However, our findings point toward an integrin-dependent platelet clearance mechanism, as platelet count and platelet survival in circulation were markedly improved in Rasa3-mutant mice after genetic deletion of the Rap GEF, CalDAG-GEFI (18), or the Rap1 target, talin1, and in mice treated with a blocking antibody against α_IIb_β_3_ integrin. Interestingly, this platelet clearance mechanism seems to occur without inducing overt platelet aggregation and thrombosis, and it occurs in the spleen and liver. In contrast, the lung is the predominant site of integrin-dependent thrombosis following thrombin-, collagen-, or immune complex–mediated platelet activation (39, 40). Thus, Rasa3 is critical to prevent spontaneous integrin activation and platelet sequestration mediated by the CalDAG-GEFI/Rap1/talin signaling module.

We hypothesize that the peri-MZ trapping of platelets occurs because of the local environment; the MZ is where blood first enters the open circulation of the spleen (41). The spleen is known to retain a pool of platelets representing up to one-third of all circulating platelets (42), but the majority of these platelets will reenter circulation through the collecting venous system. However, when preactivated platelets enter the spleen open circulation, direct interactions with the exposed matrix and cells may cause platelet adhesion. We attempted to identify the integrin(s) and ligand(s) mediating clearance of *R3^hlb/hlb^* platelets and determined that the clearance is partially dependent on talin1 and α_IIb_β_3_, but independent of α_IIb_β_3_-fibrinogen interaction required for platelet aggregation (33). Fibronectin is another candidate α_IIb_β_3_ ligand, and β_1_ integrins and their ligands, such as α_6_β_1_/laminin, may also contribute to *R3^hlb/hlb^* platelet adhesion in the spleen. We previously demonstrated increased β_1_ integrin activation in *R3^hlb/hlb^* platelets (18), and laminin is abundant in the murine spleen MZ (43). Interestingly, neither splenectomy nor macrophage depletion normalized the life span of *R3^hlb/hlb^* platelets in circulation. We observed greater liver clearance of *R3^hlb/hlb^* platelets, which was further enhanced in splenectomized mice, suggesting multiple platelet clearance mechanisms operate simultaneously to prevent prolonged circulation of preactivated platelets. In the absence of macrophages, *R3^hlb/hlb^* platelets still become trapped in the spleen peri-MZ and likely in the liver, but we did not investigate the ultimate fate of these platelets. Firmly adhered *R3^hlb/hlb^* platelets may eventually undergo apoptosis and fragment or be cleared by other immune cells such as neutrophils (44).

Our findings have important clinical implications, as they suggest that thrombocytopenia associated with increased integrin function may primarily be the result of platelet clearance, but not impaired PPF. Macrothrombocytopenia is characteristic of atypical Glanzmann’s thrombasthenia (GT; ref. 45), a platelet function disorder caused by gain-of-function (GOF) mutations in α_IIb_β_3_. MKs in atypical patients with GT exhibit abnormal morphology (46, 47), and it is generally accepted that the thrombocytopenia is caused by impaired platelet production. However, platelet survival is not typically studied in these patients. Macrothrombocytopenia was recently also reported for 2 patients with GOF mutations in RAP1B (48). Interestingly, a profound reduction in platelet survival time was reported for 1 of the individuals, suggesting that accelerated platelet clearance due to increased integrin signaling/function contributes markedly to the thrombocytopenia observed in these inherited platelet disorders. Thrombocytopenia can also occur in patients with ITP with anti-α_IIb_β_3_ antibodies (34) or patients undergoing surgery treated with α_IIb_β_3_ inhibitors (49), i.e., in acquired platelet disorders where integrin function may be altered. Some patients with ITP do not respond to treatments such as steroids or splenectomy (50), casting doubt on the notion that platelet clearance in ITP is primarily the result of Fc receptor–mediated phagocytosis in the spleen. Our studies demonstrate that anti-α_IIb_β_3_ antibody–induced platelet sequestration in the spleen resembles that observed in Rasa3-mutant mice. Furthermore, a similar pattern of splenic MZ platelet accumulation was described for a rat model of sepsis-induced thrombocytopenia (51), i.e., in a situation where a weak platelet agonist (lipopolysaccharide) induces platelet clearance. Thus, altered integrin function may also contribute to splenic platelet clearance in acquired thrombocytopenias.

In summary, our studies show that macrothrombocytopenia in *R3^hlb/hlb^* mice is due almost entirely to impaired platelet survival, with platelet production in vivo being largely intact. Rasa3-mutant platelets become trapped in the spleen and liver in a talin1/integrin-dependent manner and are likely phagocytosed by resident macrophages or other immune cells. This mechanism of integrin-dependent platelet clearance is independent of overt thrombosis. We propose that dysregulated integrin signaling may contribute to increased platelet clearance in various inherited and acquired thrombocytopenias and that a better understanding of compensatory clearance mechanisms and the hierarchal organization of such mechanisms could lead to the development of improved treatment strategies in these types of thrombocytopenic conditions.

## Methods

### Mice.

*R3^hlb/hlb^* and *R3^–/–^*;(18), *Caldaggef1^–/–^* (*Cdg1^–/–^*; ref. 19), and *Talin1^fl/fl^ Pf4-Cre^+^* (*Tln1^mKO^*; ref. 32) mice were previously described. *Tg(act-EGFP)Y01Osb* (EGFP^+^; ref. 52) mice were a gift from Hua Zhang of the University of North Carolina (UNC). Fibrinogen γ^Δ*5/*Δ5^ mice (33) and littermate controls were provided by UNC. All mice were bred in-house and on a C57BL/6J background. C57BL/6J mice obtained from The Jackson Laboratory were used as recipients for transfusion and anti-platelet antibody experiments. Splenectomy and sham operations were performed by the McAllister Heart Institute Animal Surgery Core (now the Cardiovascular Physiology and Phenotyping Core) at UNC, and mice were allowed to recover for 2 weeks before additional experiments. Both male and female mice were used for experiments.

### Cryosection IF.

Femur samples were taken and fixed with 5% paraformaldehyde and 30% sucrose and dehydrated using a graded sucrose series. Samples were then embedded in O.C.T. Compound (Tissue-Tek) and frozen on dry ice. Sections of 8 μm were cut using a Leica 2235 rotary microtome and a CryoJane Tape Transfer frozen sectioning system (Leica Biosystems). Samples were permeabilized using 0.5% Triton X-100 for 30 minutes, then probed with anti–GPIX–Alexa Fluor (AF) 488 antibody (4 μg/mL; Emfret, clone Xia.B4) to specifically label MKs/platelets and anti–CD105-AF647 antibody (2.5 μg/mL; BioLegend, clone MJ7/18) to stain the endothelium. Nuclei were stained using Hoechst (Invitrogen, Thermo Fisher Scientific). For spleen, liver, and lung, mice were sacrificed, and transcardial perfusion was performed with PBS followed by 4% formaldehyde. Organs were dissected and small pieces were immersion fixed for 4 hours in formaldehyde and then cryoprotected in 30% sucrose overnight before being embedded in O.C.T. Compound. Sections of 10 μm were cut and allowed to dry overnight. Slides were fixed with acetone, rehydrated in PBS, and incubated overnight with AF-conjugated primary antibodies (anti–GPIX-AF488 [2.5 μg/mL], anti–F4/80-AF647 [BioLegend, clone BM8, 1:500], or anti–CD31-AF647 [BioLegend, clone MEC13.3, 1:500]) or unconjugated primary antibody anti-CD209b (Invitrogen, Thermo Fisher Scientific; clone 22D1, 1:1000) followed by 1-hour incubation with anti–Armenian hamster–AF647 antibody (BioLegend, clone Poly4055, 1:2000). For organ sections from mice receiving EGFP platelet transfusions, perfusion and immersion fixation was reduced to 1% formaldehyde to prevent leakage or overfixation of cytosolic EGFP (53). The next day, slides were washed, briefly stained with Hoechst, and mounted on glass coverslips with ProLong Gold Antifade mountant (Thermo Fisher Scientific). Samples were visualized on a Leica DM4000 confocal microscope equipped with a 40× oil objective. Confocal *Z*-stacks of spleen, liver, and lung cryosections were obtained and shown as *Z* max projections.

### EM.

BM samples obtained by flushing mouse femurs with 0.1 M sodium cacodylate buffer were fixed in 2.5% glutaraldehyde and embedded in Epon as described (54). Thin sections were stained with uranyl acetate and lead citrate and examined under a JEM-2100Plus electron microscope (Jeol). MK maturation stage was classified as follows: stage (I) the earliest recognizable MKs 10–15 μm in diameter with a large nucleus, (II) cells 15–30 μm in diameter containing platelet-specific granules, and (III) mature MKs, 30–50 μm in diameter, having a well-developed DMS with clearly defined cytoplasmic territories and a PZ devoid of organelles.

### Ex vivo PPF assay (BM explants).

BM explants were obtained by flushing femurs, cutting into 1 mm pieces, and staining with anti–GPIX-AF488 antibody (10 μg/mL) for 30 minutes. Explants were then placed in a glass-bottom culture dish (MatTek) precoated overnight with fibronectin (20 μg/mL; MilliporeSigma) with Tyrode’s buffer (137 mM NaCl; 2 mM KCl; 0.3 mM NaH_2_PO_4_; 5.5 mM glucose; 5 mM N-2-hydroxyethylpiperazine-N′-2-ethanesulfonic acid; 12 mM NaHCO_3_; 2 mM CaCl_2_, pH 7.4) and maintained at 37°C in a VivaView FL Incubator Microscope (Olympus). Images were acquired every 5 minutes in differential interference contrast and GFP channels for 18 hours to visualize MKs released at the periphery of the explants. Videos were analyzed using ImageJ (NIH).

### Preparation of MKs for in vitro PPF and ploidy analysis.

BM cells were obtained by flushing femurs and tibias of mice. The cell population was enriched in mature MKs using a 1.5/3% BSA gradient under gravity (1*g*) for 45 minutes at room temperature, then incubated overnight in Tyrode’s buffer. The following day, MKs were fixed with 1% formaldehyde and labeled using anti–GPIX-AF488 antibody, and the percentage of MKs undergoing PPF was determined using a Nikon Ti-U inverted microscope (Nikon Instruments).

### 2P-IVM.

Mice were anesthetized by i.p. injection of 1.5% xylazine and 10% ketamine (10 μL/g of body mass) and placed on a heating pad. For calvarial BM imaging, a 1 cm incision was made along the midline to expose the frontoparietal skull, while carefully avoiding damage to the bone tissue. The mouse was placed on a custom metal stage equipped with a custom stereotactic holder to immobilize the head. BM vasculature was visualized by injection of tetramethylrhodamine dextran (5 μg/g BW; MilliporeSigma). MKs/platelets were visualized by injection of anti–GPIX-AF488 antibody (0.5 μg/g BW). For spleen imaging, an incision was made on the left dorsal side of the animal, and the spleen was externalized and a coverslip placed over it to provide a flat surface for imaging. Imaging was performed 18–24 hours after transfusion of EGFP^+^ platelets. RPMs were visualized by injection of anti–F4/80-AF594 antibody (2.5 μg/mouse). Images were acquired on an Olympus FV1000MPE with an Insight DeepSee IR laser (Spectra-Physics) and an upright BX-61WI microscope, using a 25× 1.05 NA water immersion objective. Images were gathered with Olympus Fluoview software, and movies were generated with Fiji ImageJ Software (Version 2.0).

### Clodronate liposome administration.

Clodronate or control (PBS) liposomes (Liposoma) were administered by i.v. injection (100 μL suspension/10 g BW) for depletion of spleen and liver macrophages. After 24 hours, liposome-treated mice were transfused with EGFP^+^ platelets. Macrophage depletion was confirmed by absence of intact F4/80^+^ macrophages in splenic cryosections.

### Platelet transfusion.

Platelets for transfusion were collected from *EGFP^+^ R3^+/+^ Cdg1^+/–^* (C) or *EGFP^+^* *R3^hlb/hlb^ Cdg1^+/–^* (*hlb*) mice. Blood was collected via the retroorbital plexus from donor mice into tubes containing PBS with low molecular weight heparin (LMWH; 30 IU/mL) using shortened heparinized glass capillaries (VWR). Whole blood was diluted with Tyrode’s buffer–Ca^2+^ and centrifuged at 130*g* for 4 minutes at RT using a centrifuge equipped with a swinging bucket rotor. The top layer of platelet-rich plasma (PRP) was collected along with some RBCs and spun at 100*g* for 5 minutes. The top layer of PRP was again collected, without disturbing the RBC pellet, incubated with prostacyclin (1 μg/mL) for 5 minutes, and then spun at 700*g* for 5 minutes. Finally, the top layer was discarded, and the platelet pellet was gently resuspended in Tyrode’s buffer–Ca^2+^. For C platelets, 1 donor per recipient was used; for *hlb* platelets, due to low platelet counts (≤20% of C), platelet pellets from 7 donor mice were pooled. Platelet concentration was determined by flow cytometry, and WT recipient mice were transfused to achieve a transfused platelet count of approximately 1 × 10^8^/mL (representing approximately 10% of normal circulating platelet count in a WT mouse). To confirm platelet counts, 50 μL blood samples were collected 20 minutes after transfusion. Transfused platelets were identified by EGFP fluorescence.

### Liver intraviral microscopy.

Liver imaging was performed on recipient mice 18–24 hours after EGFP^+^ platelet transfusion. Mice were anesthetized by i.p. injection of a ketamine/xylazine solution and redosed with ketamine (50 μL; 10%) at 30-minute intervals or if toe pinch reflex reappeared. KCs were labeled by i.v. injection of anti–F4/80-AF647 antibody (2.5 μg/mouse). The mouse was placed supine on a heated stage, abdominal hair removed with Nair, and a small incision was made in the abdominal wall just below the ribs. The mouse was then placed in the left lateral decubitus position and the left lateral lobe of the liver was guided with cotton swabs onto the coverslip of the heated stage. A thin wipe was placed over the externalized lobe and wet with sterile saline to keep the liver from drying out and to use the surface tension to maintain stability during imaging. The heated stage was then placed over a 40× oil-immersion objective on a Leica TCS SPE DMI 4000 inverted confocal microscope for intravital imaging. Videos were recorded using Leica Application Suite X (LasX) software. A total of 4–5 random fields with normal blood flow were chosen, and 5-minute videos were recorded for each field. Analysis of real-time platelet adhesion was performed using the TrackMate plugin for Fiji (55). Adhesion events were considered as platelets remaining in-frame for more than or equal to 5 frames (2.55 s/frame).

### Platelet glycosylation analysis.

Platelet receptor glycosylation was determined by binding of specific lectins: PNA recognizing Galβ3GalNAc, ECL recognizing Galβ4GlcNAc, and RCA1 recognizing Gal residues (Galβ1-4GlcNAc) (Vector Laboratories; ref. 29). Washed platelets (5 μL of 3 × 10^8^/mL) were incubated with fluorescein-conjugated lectins (25 μg/mL) and anti–GPIX-AF647 (2 μg/mL) for 10 minutes at RT, then diluted in PBS before analysis by flow cytometry. As a positive control for RCA1 lectin binding, platelets were treated with neuraminidase (100 U/mL) for 10 minutes prior to incubation with RCA1.

### Bench-aged platelet analysis.

Blood was collected in PBS/LMWH and allowed to sit on the bench at RT for 8 hours. The tubes were gently mixed every 30 minutes to avoid settling. At *t* = 0, 2, 4, and 8 hours, an aliquot was removed from each tube for flow cytometry analysis. Aliquots were stained with anti–P selectin–AF647 (2 μg/mL; BD Biosciences, clone RB40.34), JON/A-PE recognizing the activated conformation of α_IIb_β_3_ (2 μg/mL; Emfret Analytics, clone JON/A-PE), and annexin V–FITC, which binds PS exposed on the outer leaflet of the plasma membrane (5 μg/mL; provided by Sriram Krishnaswamy, University of Pennsylvania, Philadelphia, Pennsylvania, USA), and MFI was analyzed. Platelets were gated on GPIX expression.

### PNAs.

Analysis of PNAs was performed as previously described (31). Briefly, heparinized whole blood was diluted 1:6 in PBS and incubated with anti–Ly6G-AF647 (BioLegend, clone 1A8) and anti–GPIX-AF488 antibodies (2 μg/mL each) for 15 minutes. Samples were then mixed 1:4 with Phosflow Lyse/Fix buffer (BD Biosciences) for 30 minutes at 37°C before analysis by flow cytometry. Neutrophils were gated on Ly6G intensity, and PNAs were identified as GPIX^+^ neutrophils.

### Platelet counts.

Blood (50 μL) was obtained and diluted 1:1 with PBS/LMWH. A 2 μL aliquot of whole blood was stained with anti–GPIX-AF488 antibody (2 μg/mL) for 10 minutes at room temperature, diluted in PBS, and analyzed by flow cytometry. Platelets were gated on forward scatter height (FSC-H) and GPIX intensity.

### Platelet life span assay.

Mice were injected retro-orbitally (R.O.) with 2.5 μg anti–GPIX-AF488 antibody to label all circulating platelets, and blood was collected 10 minutes later for *t* = 0 platelet count. Blood was then collected every 24 hours, stained with anti–GPIbα-PE antibody (2 μg/mL; Emfret Analytics, clone Xia.G5), and then analyzed by flow cytometry. All circulating platelets were gated on GPIbα intensity, and the percentage of AF488^+^ platelets remaining was determined.

### Antibody administration.

To induce thrombocytopenia, mice were injected R.O. with anti-GPIbα platelet-depleting antibody (Emfret Analytics, clone R300) or anti–α_IIb_β_3_ integrin antibody (BD Biosciences, clone MWReg30) at 2 μg/g BW. Platelet counts were determined 5 minutes before and 30 minutes after antibody injection, mice were then sacrificed and perfused, and organs were collected for sectioning. To block α_IIb_β_3_ function, mice were injected R.O. with 75 μg anti–α_IIb_β_3_ F(ab′)_2_ antibody (clone 4H5) every 24 hours. Inhibition of α_IIb_β_3_ was confirmed using a whole-blood platelet aggregation assay.

### Statistics.

Data are shown as mean ± SEM unless otherwise noted. For statistical analysis, GraphPad Prism software (version 9.2 for Windows) was used. Comparisons between 2 groups were done using unpaired, 2-tailed Student’s *t* test. Comparisons between more than 2 groups were done using 1-way or 2-way ANOVA with Bonferroni’s post hoc test for multiple comparisons. *P* < 0.05 was considered statistically significant.

### Study approval.

All experimental protocols were approved by the UNC Institutional Animal Care and Use Committee.

## Author contributions

RHL and DG designed and performed experiments, analyzed and interpreted data, and drafted the manuscript. GLH, JPK, and PAK performed experiments. FP performed experiments and analyzed and interpreted data. BN and MJF contributed critical reagents. CG contributed substantial editing of the manuscript. CC performed experiments and analyzed and interpreted data. AE performed experiments and analyzed and interpreted data. WB designed and supervised experiments, interpreted data, and drafted the manuscript. All authors read and approved the manuscript. RHL and DG both contributed significantly to data acquisition and analysis. RHL is listed first of the co–first authors for performing the majority of the manuscript drafting, editing, revising, and formatting following acceptance.

## Supplementary Material

Supplemental data

Supplemental video 1

Supplemental video 2

Supplemental video 3

Supplemental video 4

Supplemental video 5

Supplemental video 6

Supplemental video 7

Supplemental video 8

Supplemental video 9

## Version 1 03/15/2022

In-Press Preview

## Version 2 04/22/2022

Electronic publication
