# Supplementary material for: Rasa3 deficiency minimally affects thrombopoiesis but promotes severe thrombocytopenia due to integrin-dependent platelet clearance
Source: JCI Insight. 2022 Apr 22;7(8):e155676. doi: 10.1172/jci.insight.155676 (PMC9089782; doi:10.1172/jci.insight.155676)
Supplement: Supplemental data [file jciinsight-7-155676-s090.pdf]

Supplemental Figures

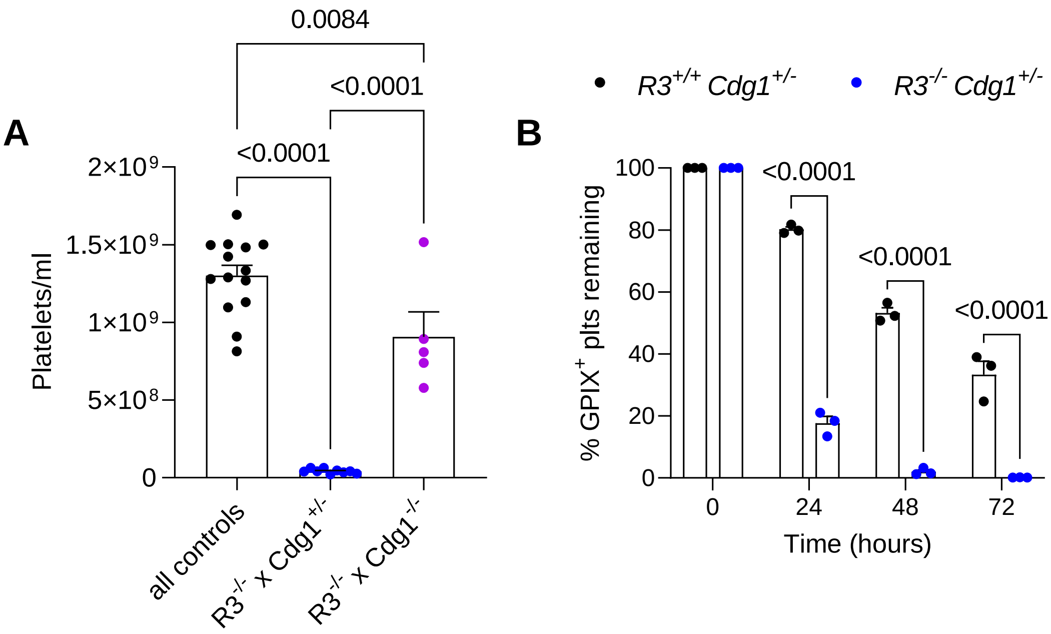

Supplemental Figure 1: Severely reduced platelet counts and platelet lifespan in *R3$^{-/-}$ x Cdg1$^{+/-}$* mice. (**A**) Circulating platelet counts were determined by flow cytometry using whole blood from *R3$^{-/-}$ x Cdg1$^{+/-}$*, *R3$^{-/-}$ x Cdg1$^{-/-}$* and mixed control mice (n=5-14). (**B**) Mice were injected I.V. with anti-GPIX-AF488 antibody (2.5 µg/mouse) on day 0 to label all circulating platelets, and then the percentage of GPIX-AF488^{+} platelets remaining was determined every 24 hrs by flow cytometry (n=3). Data shown as mean ± SEM. Statistical significance was determined using one-way (A) or two-way (B) ANOVA with Bonferroni correction for multiple comparisons.

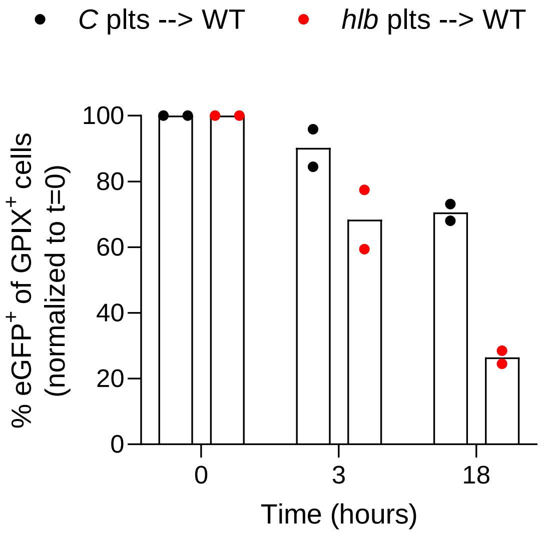

Supplemental Figure 2: Rapid clearance of transfused eGFP^{+} *R3$^{hlb/hlb}$* x *Cdg1$^{+/-}$* platelets in WT recipient mice. Platelets from eGFP^{+} *R3$^{+/+}$ x Cdg1$^{+/-}$* (C) or eGFP^{+} *R3$^{hlb/hlb}$ x Cdg1$^{+/-}$* (*hlb*) mice were transfused into recipient WT mice (n=2). The percentage of eGFP^{+} platelets of all GPIX^{+} platelets was determined at 20 mins (t=0), 3 hrs and 18 hrs after transfusion, and normalized to t=0.

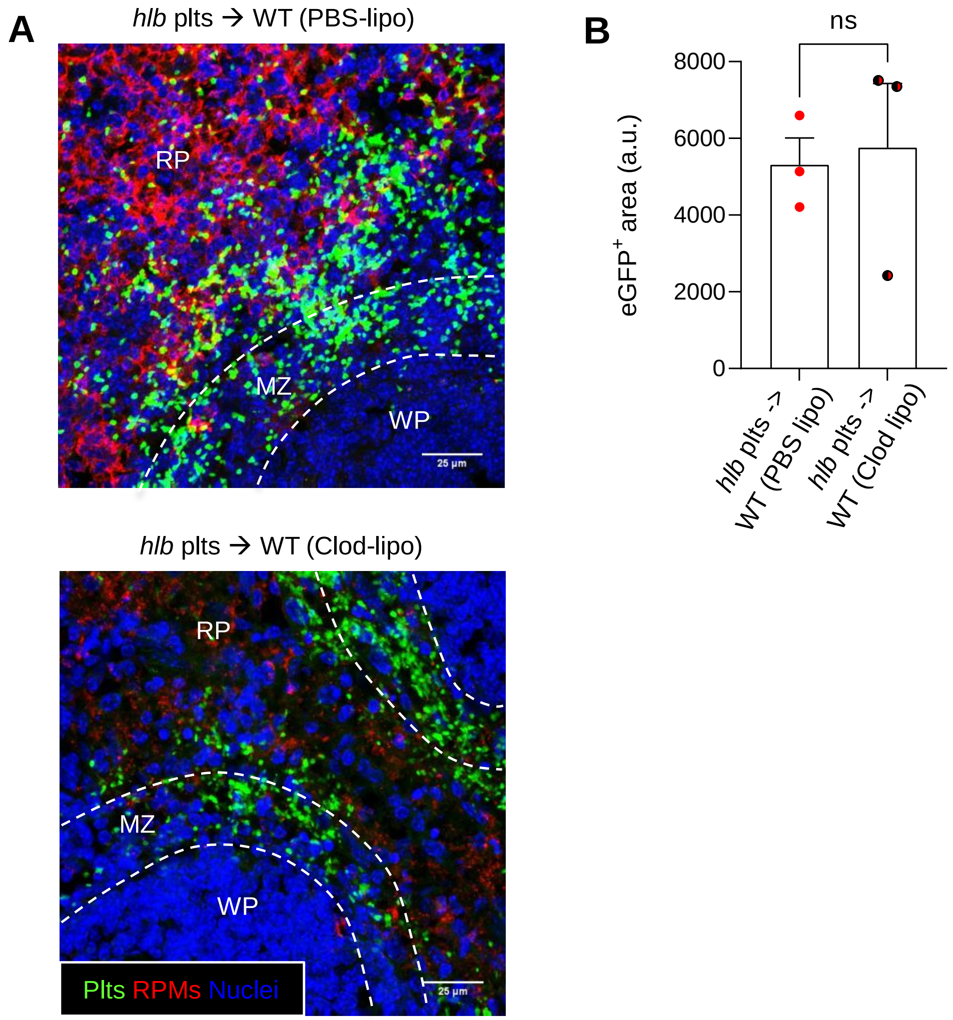

Supplemental Figure 3: Macrophage depletion does not impair splenic sequestration of *Rasa3*$^{hlb/hlb}$ platelets. (**A**) IF images of spleen cryosections from WT mice injected with either PBS (PBS-lipo) or Clodronate-containing (Clod-lipo) liposomes, and subsequently transfused with eGFP^{+} *R3*$^{hlb/hlb}$ *x Cdg1*$^{+/-}$ (*hlb*) platelets (24 hrs post-liposomes). Cryosections were stained for RPMs with anti-F4/80-AF647 (red) and nuclei with DAPI (blue), and platelets were visualized by eGFP intensity (green). Scale bars equal 25 µm. (**B**) Platelet (eGFP^{+}) area was determined in spleen cryosections using Fiji, and expressed as area in arbitrary units (a.u.) (n=3). Data shown as mean ± SEM. Statistical significance was determined using Student's t-test, ns=not significantly different.

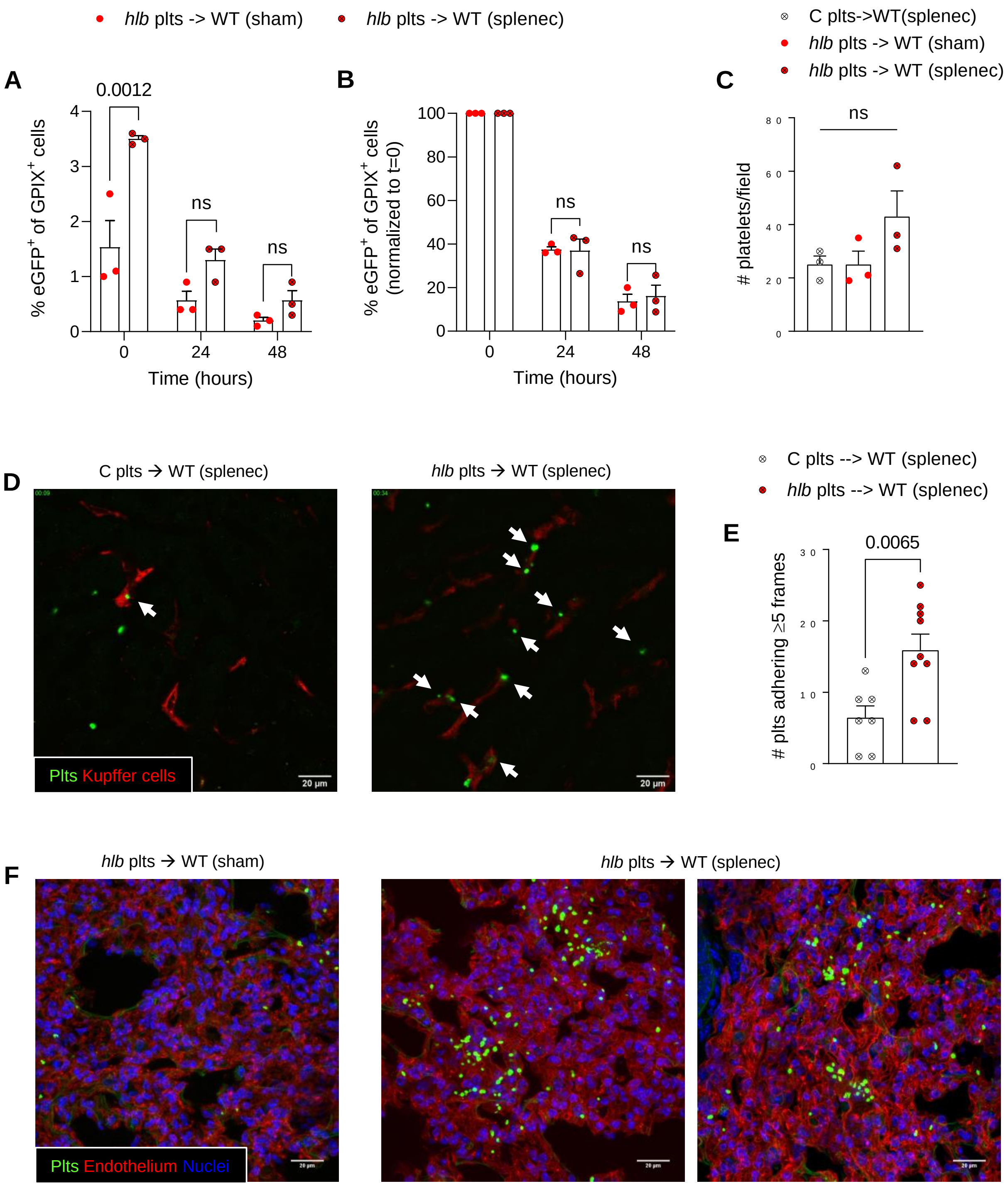

Supplemental Figure 4: Secondary liver and lung clearance of *Rasa3*$^{hlb/hlb}$ platelets compensates in the absence of the spleen. (**A**) Recovery and survival of transfused eGFP^{+} *hlb* platelets in splenectomized (splenec) or sham-operated (sham) WT recipient mice at 20 mins (t=0), 24 hrs and 48 hrs post-transfusion (n=3). (**B**) Data from (a) normalized to t=0 for each genotype. (**C**) Platelets were quantified in immunostained liver cryosections from WT (splenec) or WT (sham) mice transfused with C or *hlb* platelets

(n=3 mice). (**D**) Still frames from time-lapse confocal videos acquired in the liver of WT (splenec) recipients transfused with eGFP$^+$ C or *hlb* platelets 18-24 hrs post platelet transfusion. Platelets were visualized by eGFP fluorescence (green), and Kupffer cells were labeled *in vivo* by I.V. administration of anti-F4/80-AF647 antibody (2.5 μg/mouse; red). Scale bars equal 20 μm. Arrowheads denote adherent platelets in contact with Kupffer cells within liver sinusoids. (**E**) Quantification of platelet adhesion in liver sinusoids from intravital confocal videos (3-5 different 5-min videos were analyzed for n=2 mice per group). (**F**) Representative images of lung cryosections from WT (sham) or WT (splenec) mice transfused with *hlb* platelets. Platelets are visualized by eGFP fluorescence (green), endothelium was stained with anti-CD31-AF647 antibody (red), and nuclei with DAPI (blue). Scale bars equal 20 μm. Data shown as mean ± SEM. Statistical significance was determined using two-way (A,B) or one-way (C) ANOVA with Bonferroni correction for multiple comparisons, or Student's t-test (E), ns=not significantly different.

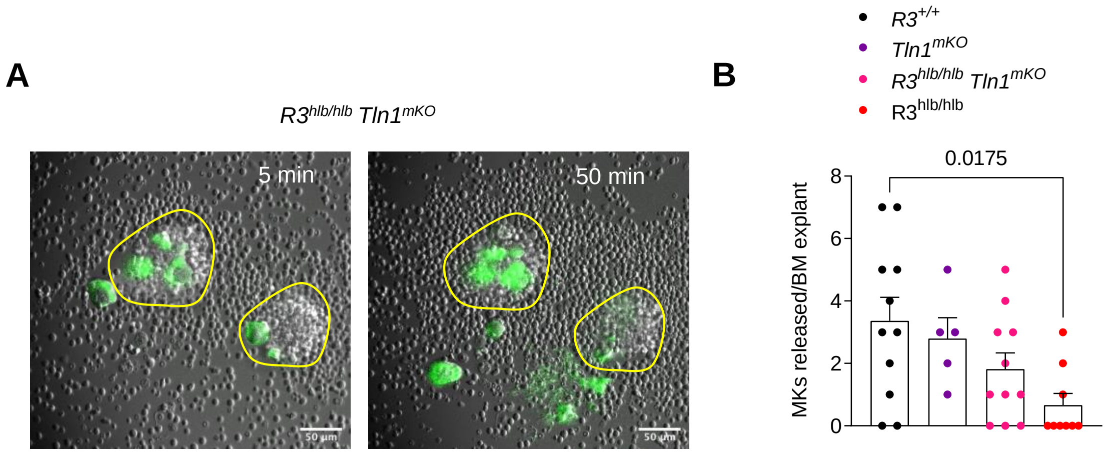

Supplemental Figure 5: Genetic deletion of Talin1 partially rescues impaired ex vivo proplatelet formation in *Rasa3*$^{hlb/hlb}$ MKs. (**A**) Representative images of ex vivo proplatelet-forming MKs from *R3*$^{hlb/hlb}$ *x Tln1*^{mKO} mice taken at the indicated time points after start of imaging. Samples were stained with anti-GPIX-AF488 to label MKs/platelets (green). Scale bars equal 50 µm. Yellow lines delimit BM pieces. (**B**) MKs released from BM explants, expressed as number of MKs released per explant in each field of view (n=5-11). Data shown as mean ± SEM. Statistical significance was determined using one-way ANOVA with Bonferroni correction for multiple comparisons.

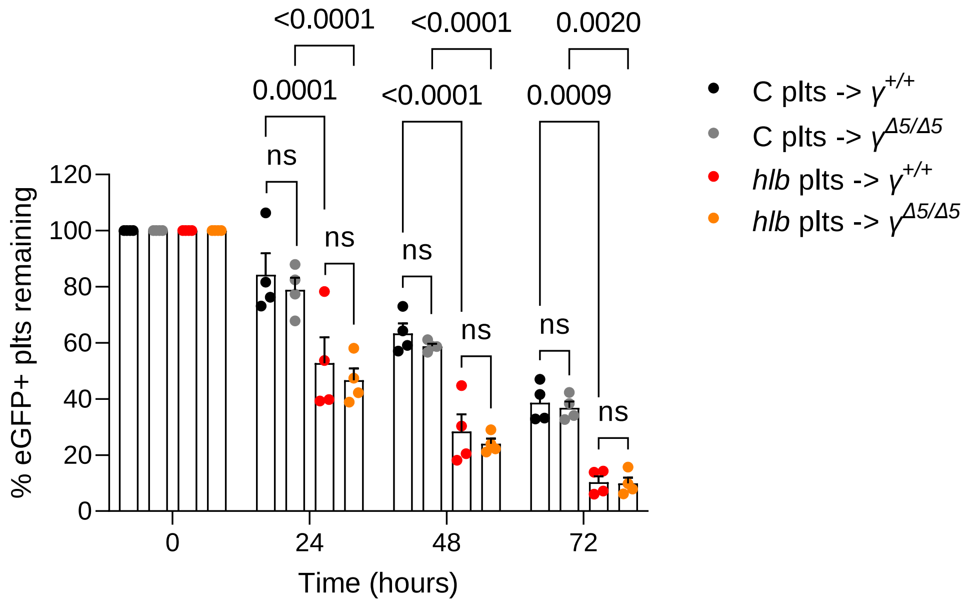

Supplemental Figure 6: Transfused *hlb* platelets are cleared equally fast in fibrinogen $\gamma^{\Delta5/\Delta5}$ mice. Platelets from eGFP^{+} *R3*$^{+/+}$ *x Cdg1*$^{+/-}$ (C) or eGFP^{+} *R3*$^{hlb/hlb}$ *x Cdg1*$^{+/-}$ (*hlb*) mice were transfused into recipient fibrinogen $\gamma^{\Delta5/\Delta5}$ or littermate control mice (n=4). The percentage of eGFP^{+} platelets of all GPIX^{+} platelets was determined at 20 mins (t=0) and every 24 hrs thereafter, and normalized to t=0. Data shown as mean ± SEM. Statistical significance was determined using two-way ANOVA with Bonferroni correction for multiple comparisons, ns=not significantly different.

Supplemental Videos

Supplemental Video 1: 3D reconstruction of confocal Z stack from *R3*$^{+/+}$ BM explant. MKs and platelets are labeled in green, endothelium in red, nuclei in blue.

Supplemental Video 2: 3D reconstruction of confocal Z stack from *R3*$^{hlb/hlb}$ BM explant. MKs and platelets are labeled in green, endothelium in red, nuclei in blue.

Supplemental Video 3: Time lapse imaging of ex vivo proplatelet formation in BM explants from a *R3*$^{+/+}$ mouse. MKs are labeled in green, overlaid with brightfield.

Supplemental Video 4: Time lapse imaging of ex vivo proplatelet formation in BM explants from a *R3*$^{hlb/hlb}$ mouse. MKs are labeled in green, overlaid with brightfield.

Supplemental Video 5: Time lapse spleen IVM using 2-photon microscopy in a WT mouse transfused with eGFP^{+} *R3*$^{+/+}$ x *Cdg1*$^{+/-}$ ("C") platelets. Platelets are green, red pulp macrophages are labeled in red.

Supplemental Video 6: Time lapse spleen IVM using 2-photon microscopy in a WT mouse transfused with eGFP^{+} *R3*$^{+/+}$ x *Cdg1*$^{+/-}$ ("*hlb*") platelets. Platelets are green, red pulp macrophages are labeled in red.

Supplemental Video 7: Time lapse liver IVM using laser scanning confocal in a WT mouse transfused with eGFP^{+} *R3*$^{+/+}$ x *Cdg1*$^{+/-}$ ("C") platelets. Platelets are green, Kupffer cells are labeled in red.

Supplemental Video 8: Time lapse liver IVM using laser scanning confocal in a WT mouse transfused with eGFP^{+} *R3*$^{+/+}$ x *Cdg1*$^{+/-}$ ("*hlb*") platelets. Platelets are green, Kupffer cells are labeled in red.

Supplemental Video 9: 3D reconstruction of confocal Z stack from *R3*$^{hlb/hlb}$ *x Tln1*^{mKO} BM explant. MKs and platelets are labeled in green, endothelium in red, nuclei in blue.
